# Electronic Structure and Band Alignments of Various Phases of Titania Using the Self-Consistent Hybrid Density Functional and DFT+*U* Methods

**DOI:** 10.3389/fchem.2019.00047

**Published:** 2019-02-07

**Authors:** Won June Kim, Myung Hoon Han, Sébastien Lebègue, Eok Kyun Lee, Hyungjun Kim

**Affiliations:** ^1^CNRS, LPCT, UMR 7019, Université de Lorraine, Vandœuvre-lès-Nancy, France; ^2^Department of Chemistry, Korea Advanced Institute of Science and Technology (KAIST), Daejeon, South Korea

**Keywords:** photocatalyst, titania, band alignment, self-consistent GGA+*U*, self-consistent hybrid functional

## Abstract

To understand, and thereby rationally optimize photoactive interfaces, it is of great importance to elucidate the electronic structures and band alignments of these interfaces. For the first-principles investigation of these properties, conventional density functional theory (DFT) requires a solution to mitigate its well-known bandgap underestimation problem. Hybrid functional and Hubbard *U* correction are computationally efficient methods to overcome this limitation, however, the results are largely dependent on the choice of parameters. In this study, we employed recently developed self-consistent approaches, which enable non-empirical determination of the parameters, to investigate TiO_2_ interfacial systems—the most prototypical photocatalytic systems. We investigated the structural, electronic, and optical properties of rutile and anatase phases of TiO_2_. We found that the self-consistent hybrid functional method predicts the most reliable structural and electronic properties that are comparable to the experimental and high-level GW results. Using the validated self-consistent hybrid functional method, we further investigated the band edge positions between rutile and anatase surfaces in a vacuum and electrolyte medium, by coupling it with the Poisson-Boltzmann theory. This suggests the possibility of a transition from the straddling-type to the staggered-type band alignment between rutile and anatase phases in the electrolyte medium, manifested by the formation of a Stern-like layer at the interfaces. Our study not only confirms the efficacy of the self-consistent hybrid functional method by reliably predicting the electronic structure of photoactive interfaces, but also elucidates a potentially dramatic change in the band edge positions of TiO_2_ in aqueous electrolyte medium which can extensively affect its photophysical properties.

## Introduction

Titania (TiO_2_) is one of the most prototypical materials, utilized in a wide range of photocatalytic and photovoltaic applications (Diebold, 2003; Thompson and Yates, 2006; Fujishima et al., 2008; Henderson, 2011; Schneider et al., 2014). Depending on octahedron connectivity, TiO_2_ has several polymorphs, among which the rutile phase is known to be the ground state and the anatase phase is usually regarded as a better photocatalyst (Luttrell et al., 2014). For mechanistic investigations of the photophysical processes, toward optimization of the photocatalytic or photovoltaic activity, it is important to understand the electronic structures, such as bandgap properties and band edge alignments of the various TiO_2_ phases. However, due to the sensitivity of electronic structures, to the surface state (e.g., defects or molecular adsorptions, particle size, dielectric environment, etc.) (Diebold, 2003; Yu et al., 2003; Stevanovic et al., 2012; Ronca et al., 2013; Amano et al., 2016; Macounová et al., 2017), a complete understanding of the electronic and optical properties of TiO_2_ remains elusive, despite a number of experimental and theoretical studies that have been conducted. For example, the relative band edge positions between the rutile and anatase phases are still under debate (Li and Gray, 2007; Scanlon et al., 2013; Zhang et al., 2014; Nolan et al., 2016).

First-principles based density functional theory (DFT) is a total energy theory, which provides a wealth of understanding on the electronic structures of materials, with a reasonable computational cost. However, the (semi-)local approximation of the Kohn-Sham (KS) DFT in describing the exchange-correlation (XC) energy invokes an inevitable problem, in which the bandgaps of semiconductors and insulators are significantly underestimated. The fundamental origin of this problem lies in the fact that the total energy vs. the number of electrons obtained with a (semi-)local XC functional, is not a series of linear segments between integer numbers (Sham and Schlüter, 1983; Anisimov et al., 1991).

To resolve the bandgap underestimation problem of KS-DFT, many theoretical advances have been achieved, including the weighted density approximation (Alonso and Girifalco, 1978), Hubbard *U* correction (DFT+*U*) method (Anisimov et al., 1991), self-interaction correction method (Perdew and Zunger, 1981), screened exchange approximation (Bylander and Kleinman, 1990), optimized effective potentials (Grüning et al., 2006), generalized Kohn-Sham (GKS) scheme (Seidl et al., 1996), meta-GGA potentials (Tao et al., 2003), and hybrid functionals (Becke, 1993). In addition to these methods, quasiparticle GW approximation (Hedin, 1965) is regarded as the most reliable and formally accurate method in terms of predicting exact band edge positions of the valence band maximum (VBM) and the conduction band minimum (CBM). However, it also negatively affected by expensive computational cost, which prohibits its routine application in large-scale systems such as surfaces or nanoparticles. On the other hand, the hybrid functional or DFT+*U* methods provide a more computationally efficient manner to accurately describe the electronic structure (Anisimov et al., 1991; Becke, 1993). By including a portion of the nonlocal Hartree-Fock (HF) exchange or an additional Hubbard-like term, the hybrid functional or DFT+*U* methods, respectively, reduce the self-interaction error of conventional DFT. However, these methods require an empirical parameter to determine either a HF mixing ratio or a Hubbard *U* parameter.

To avoid such empiricism in the hybrid functional or DFT+*U* methods, self-consistent approaches have recently been proposed for the non-empirical determination of the HF mixing ratio (Skone et al., 2014) or the Hubbard *U* parameter (Cococcioni and de Gironcoli, 2005). The self-consistent hybrid (sc-hybrid) functional method is based on the simplified form of the many-body self-energy under the static approximation, called the static Coulomb hole plus screened exchange (COHSEX), which enables a relation of the HF mixing ratio with the inverse macroscopic dielectric constant (Skone et al., 2014). Based on the linear-response property of the total energy with respect to the occupation number, the Hubbard *U* parameter can also be determined in a self-consistent manner (Cococcioni and de Gironcoli, 2005).

In this study, we investigated the electronic structures and band edge positions of the rutile and anatase phases of TiO_2_ by means of these recently developed self-consistent hybrid functional and DFT+*U* methods. In comparison with the conventional DFT results based on the generalized gradient approximation (GGA) of the XC energy, and with the GW results from previous studies, we assessed the reliability of the self-consistent methods by describing the structural, electronic, and optical properties of bulk rutile and anatase TiO_2_. We then investigated surface band alignment in rutile (110) and anatase (101) surfaces. Further, as most interesting photocatalytic reactions (e.g., water splitting) occurred in water, we examined the band edge positions in a vacuum and aqueous environment, where the solvation effect was implicitly modeled using the Poisson-Boltzmann theory (Mathew et al., 2014; Mathew and Hennig, 2016).

## Methods

### Self-Consistent Approaches in Hybrid Functional and GGA+*U* Methods

Hybrid functional (Becke, 1993) includes a portion of the HF exchange energy in addition to the (semi-)local DFT XC energy, where the mixing ratio is controlled by the parameter α. For the particular GGA functional chosen by Perdew, Burke, and Ernzerhof (PBE) (Perdew et al., 1996a), the hybrid PBE functional (PBEh) of the XC energy is written as (Perdew et al., 1996b):

$$E_{\text{xc}}^{\text{PBEh}}=\alpha E_{\text{x}}^{\text{HF}}+\left(1-\alpha \right)E_{\text{x}}^{\text{PBE}}+E_{\text{c}}^{\text{PBE}}$$

The choice of α = 0.25 gives the standard PBE0 functional, which is best fitted to yield the atomization energies of typical molecules (Adamo and Barone, 1999). More recent studies have related the parameter α with the inverse static dielectric constant of the system (ε_∞_) by comparing the HF exchange with the screened Coulomb interaction (Marques et al., 2011). Then, since the ε_∞_ is dependent on the choice of α, a self-consistent scheme was suggested to determine α without empiricism (Skone et al., 2014). From the initial guess of α_*in*_ = 0.25, self-consistency could be achieved by repeating the calculation of ε_∞_ using the PBEh functional with α_*in*_ as an input parameter, until the difference between α_*in*_ and the resultant $\alpha_{out}=\frac{1}{\varepsilon_{\infty }}$ is smaller than a specified threshold. We hereafter term the self-consistent PBE0 functional method scPBE0.

On the other hand, DFT+*U* (Anisimov et al., 1991) solves the problem of the unphysical curvature by adding the correction of a Hubbard-like interaction for the atom *I* with occupancy of its localized orbital *n*^*Iσ*^:

$$E_{\text{DFT}+U}\left[n\left(r\right)\right]=E_{\text{DFT}}\left[n\left(r\right)\right]+E_{\text{Hub}}\left[\left\{n_{m}^{I\sigma }\right\}\right]-E_{\text{DC}}\left[\left\{n^{I\sigma }\right\}\right]$$

where the last term, $E_{\text{DC}}\left[\left\{n^{I\sigma }\right\}\right]$, is subtracted to avoid double counting since it is included in both the Hubbard term and the DFT energy. Usually, as a practical choice, a simplified rotationally invariant version introduced by Dudarev et al. is employed (Dudarev et al., 1998):

$$\begin{array}{l} E_{U}\left[\left\{n_{mm^{\prime }}^{I\sigma }\right\}\right]=E_{\text{Hub}}\left[\left\{n_{mm^{\prime }}^{I\sigma }\right\}\right]-E_{\text{DC}}\left[\left\{n^{I\sigma }\right\}\right]\;\\ \;=\frac{U_{\text{eff}}}{2}\sum_{I}\sum_{m,\sigma }\left\{n_{mm}^{I\sigma }-\sum_{m^{\prime }}n_{mm^{\prime }}^{I\sigma }n_{m^{\prime }m}^{I\sigma }\right\} \end{array}$$

where the term *U*_eff_ = *U* − *J* accounts for on-site Coulomb repulsion and a mimicked effects of exchange interaction.

Cococcioni and de Gironcoli suggested a self-consistent approach to determine *U*_eff_ using linear response theory (Cococcioni and de Gironcoli, 2005). In this approach, the eigenvalue shift, α_*I*_, to localize (or delocalize) the occupation *n*_*I*_ of the Hubbard site *I* is introduced to the total energy of a constrained system:

$$E\left[\left\{\alpha_{I}\right\}\right]=\underset{n(r)}{\min}\left\{E\left[n\left(r\right)\right]+\sum_{I}\alpha_{I}n_{I}\right\}$$

In the case of molecules or solid systems, the non-linear behavior of total energy as a function of the number of electrons is also induced by the rehybridization of localized orbitals. The calculation of the constrained total energy, by fixing the non-interacting KS potential, is therefore also needed, to subtract this rehybridization effect:

$$E^{\text{KS}}\left[\left\{\alpha_{I}\right\}\right]=\underset{n(r)}{\min}\left\{E^{\text{KS}}\left[n\left(r\right)\right]+\sum_{I}\alpha_{I}^{\text{KS}}n_{I}\right\}$$

Then, *U*_eff_ can be determined as $U_{\text{eff},\;I}={\left(\chi_{0}^{-1}-\chi^{-1}\right)}_{II}$ from the interacting and non-interacting density response functions of the system with respect to these constrained problems:

$$\begin{array}{l} \;\chi_{IJ}=\frac{\partial^{2}E}{\partial \alpha_{I}\partial \alpha_{J}}=\frac{\partial n_{I}}{\partial \alpha_{J}}\;\\ \chi_{IJ}^{\text{KS}}=\frac{\partial^{2}E^{\text{KS}}}{\partial \alpha_{I}^{\text{KS}}\partial \alpha_{J}^{\text{KS}}}=\frac{\partial n_{I}}{\partial \alpha_{J}^{\text{KS}}} \end{array}$$

The response functions can be calculated as follows: we first obtained a well-converged self-consistent potential with zero perturbation (α_*I*_ = 0), and then performed both non-self-consistent and self-consistent calculations under the KS potential by applying a small potential shift (non-zero α_*I*_). From the non-self-consistent and self-consistent calculations, we obtained the non-interacting and interacting response functions, respectively.

By iteratively calculating the *U*_eff_ and response functions until *U*_eff_ converges with a specified threshold, we obtained the self-consistent value of *U*_eff_. In the latter part of this manuscript, we will drop the subscript from *U*_eff_ for brevity (hereafter referred to simply as *U*).

### Computational Details

We performed DFT calculations of bulk rutile TiO_2_, bulk anatase TiO_2_, and their corresponding surface slab models using the projector augmented wave (PAW) method as implemented in the Vienna Ab-initio Simulation Package (VASP) (Kresse and Furthmüller, 1996; Kresse and Joubert, 1999). A plane-wave expansion of 500 eV was used. The electronic iterations were continued until the energy difference from the previous step became smaller than 10^−7^ eV, and the geometry optimization was iterated until all atomic forces became smaller than 0.02 eV/Å. We optimized the bulk structures using the PBE functional (Perdew et al., 1996a) with the Γ-centered k-mesh, with grid size of 0.4 Å^−1^ (6 × 6 × 4 and 5 × 5 × 2 for rutile and anatase phases, respectively). We then employed these PBE-relaxed geometries to obtain the α parameters of scPBE0 and the self-consistent *U* parameters.

To obtain the α of scPBE0 for each phase, we calculated the static dielectric constants using the perturbation expansion after discretization (PEAD) method (Nunes and Gonze, 2001; Souza et al., 2002). Since convergence of the dielectric constant is slower than that of total energy, we used the grid size of 0.3 Å^−1^ for the k-mesh in the PEAD calculations.

We employed Quantum Espresso (QE) code (Giannozzi et al., 2009, 2017) to obtain the self-consistent *U* parameters, while all other calculations were performed using VASP. This was due to the lack of implementation of the calculation of the self-consistent *U* parameters in the official VASP code. During QE calculations, we used PAW potentials and the PBE functional in a manner analogous to that used for the VASP calculations. We further confirmed the consistency of the calculation results from two different codes by comparing the equilibrium lattice parameters, where the QE calculation used a plane-wave kinetic energy cutoff of 100 Ry and the same grid spacing to the VASP calculations (see Table 1). To minimize the artifact due to the periodic images of the density perturbation, we used 2 × 2 × 2 supercells to obtain carefully converged response functions.

**Table 1:** Structural properties, bandgaps, and static dielectric constants of bulk rutile and anatase TiO${}_{2}^{\text{a}}$.

**Method**	***a* (Å)**	***c* (Å)**	**V (Å^**3**^)**	**B (GPa)**	**ΔE_**gap**_ (eV)**	**ε^∞^**
**RUTILE**						
PBE	4.64 (4.65)^b^	2.97 (2.97)^b^	64.0	193	1.84	7.61
scPBE+*U*	4.65	2.99	64.9	197	2.09	7.13
PBE0	4.58	2.95	61.7	230	4.19	5.98
scPBE0	4.60	2.95	62.5	215	3.25	6.55
Exp.	4.59^c^	2.96^c^	62.4	211^d^	3.30^f^	7.37^h^
**ANATASE**						
PBE	3.80 (3.80)^b^	9.72 (9.73)^b^	140	169	2.12	6.60
scPBE+*U*	3.83	9.72	142	169	2.37	6.14
PBE0	3.76	9.60	136	197	4.46	5.25
scPBE0	3.77	9.62	137	189	3.75	5.58
Exp.	3.78^c^	9.50^c^	136	178^e^	3.47^g^	5.70^i^

a *a, c, V, B, ΔE_gap_, and ε^∞^ are the lattice parameters a and c, equilibrium volume, bulk modulus, electronic band gap, and static dielectric constant, respectively*.

b *Calculated lattice parameters using Quantum Espresso code*.

c *Burdett et al. (1987)*.

d *Ming and Manghnani (1979)*.

e *Dubrovinsky et al. (2001)*.

f *Tezuka et al. (1994)*.

g *Baldini et al. (2017)*.

h *Traylor et al. (1971)*.

i *Hosaka et al. (1997)*.

Using the convergence criterion of 0.01, we determined both the self-consistent α of the scPBE0 and the self-consistent *U* parameter of the PBE+*U*. Using these self-consistent parameters, we then optimized the bulk structures and surface slab models of rutile and anatase TiO_2_ phases. Both rutile (110) and anatase (101) slabs were composed of 6 TiO_2_ layers, and the middle two layers were kept fixed during the geometry relaxation to mimic the bulk structure. The lattice parameters of the slab models were taken from those of bulk structures which were optimized using the corresponding level of theory. In addition to the scPBE0 and PBE+*U*, which uses the self-consistent *U* parameter (namely, scPBE+*U*), we further performed conventional PBE and PBE0 calculations for the sake of comparison.

## Results and Discussion

### Self-Consistent Parameters

**Figure 1:**
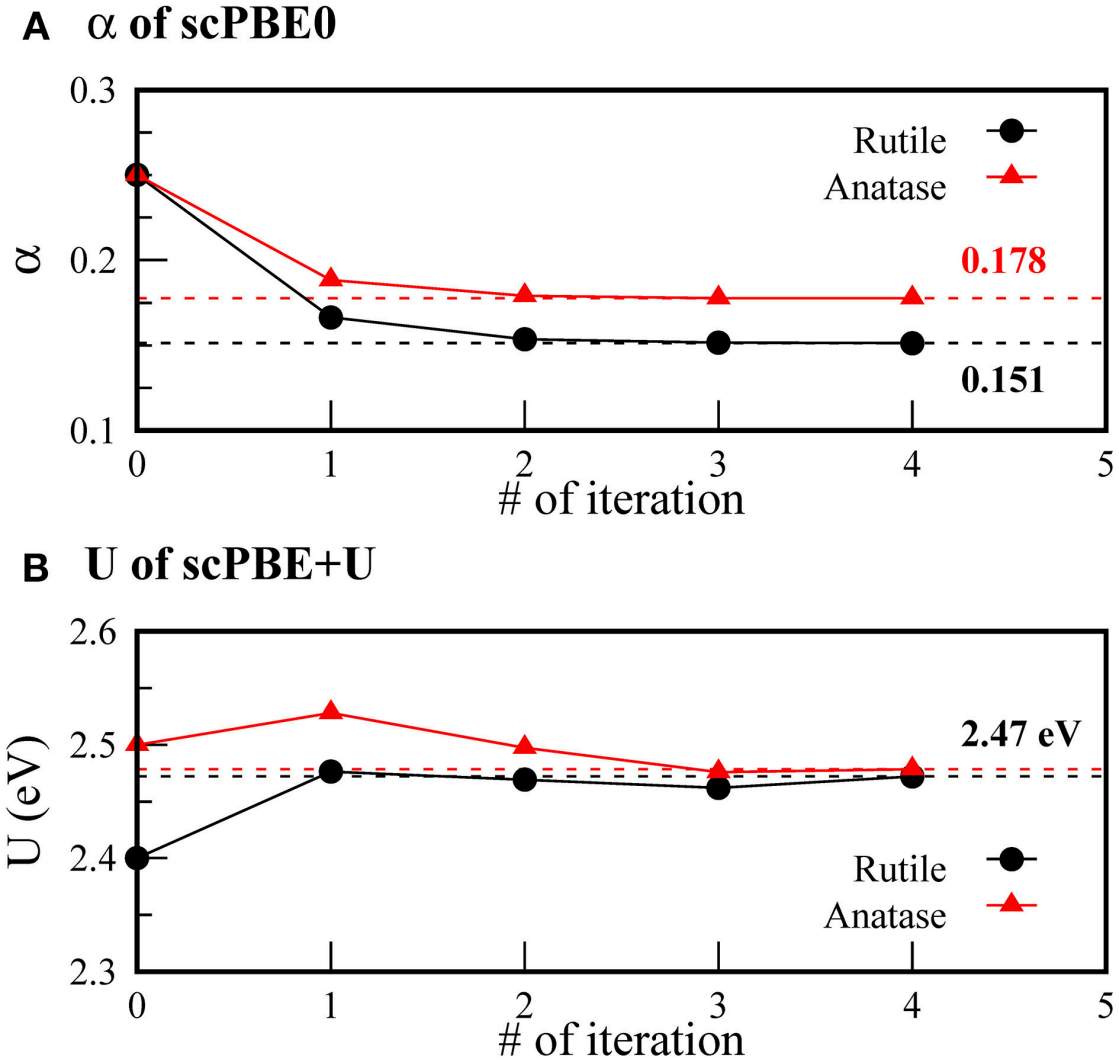
Self-consistent convergence of **(A) α** of scPBE0 and **(B)** *U* of scPBE+*U* during iterations. Black circles and red triangles denote rutile and anatase phases, respectively. The converged values are indicated by the dashed lines.

Figure 1 shows the evolution of the self-consistent parameters α and *U* during iteration. For scPBE0, we obtained α values of 0.151 and 0.178 for the rutile and anatase TiO_2_, respectively. Our α value for the rutile phase is slightly different from the self-consistent value determined by He and Franchini, 0.142 (He and Franchini, 2017). The difference was ascribed to the use of slightly different geometries optimized using different XC functionals. Our value was further compared with the empirical value of 0.159, determined to reproduce the experimental bandgap.

We obtained the same self-consistent *U* values of 2.47 eV for both the rutile and anatase phases. Our value is smaller than the frequently used empirical value of 4.20 eV determined to reproduce experimental spectroscopic data (Morgan and Watson, 2007; Araujo-Lopez et al., 2016).

### Structural and Electronic Properties of Rutile and Anatase TiO_2_

**Figure 2:**
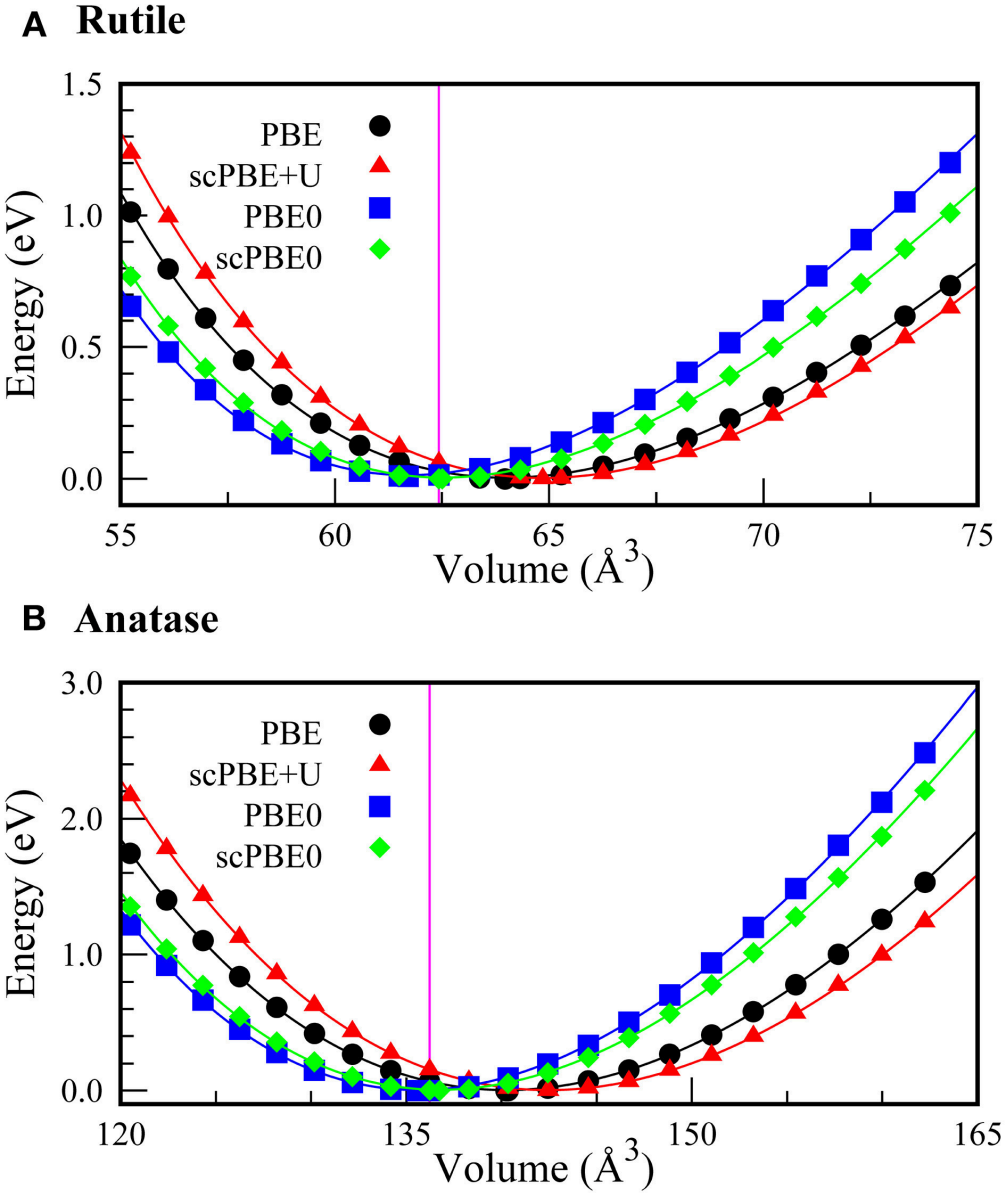
Equations of state (EoS) for **(A)** rutile and **(B)** anatase phases of TiO_2_ calculated using PBE (black circles), scPBE+*U* (red triangles), PBE0 (blue squares), and scPBE0 (green diamonds) methods. The vertical magenta lines represent the experimental volumes of the two phases.

Using the self-consistently determined values of α and *U*, we investigated the equations of state (EoS) of the rutile and anatase phases of TiO_2_. Figure 2 shows the EoS of the rutile and anatase phases calculated using PBE, scPBE+U, PBE0, and scPBE0 methods. The lattice parameters, equilibrium volumes, and bulk moduli are shown in Table 1.

We found that PBE and scPBE+*U* both overestimated the equilibrium volume and underestimated the bulk modulus, while the hybrid functionals showed better agreement with experimental values. Compared to the conventional PBE0, the self-consistent method of scPBE0 demonstrated a slight improvement.

Table 1 also shows the bandgaps and dielectric constants of rutile and anatase TiO_2_ calculated using various DFT methods. Unsurprisingly, PBE underestimated the bandgaps. However, even after the inclusion of the Hubbard *U* correction, the scPBE+*U* still significantly underestimated the bandgaps, implying that the self-consistently determined *U* value was not large enough to reproduce the experimental bandgaps. Indeed, a previous study has demonstrated that a fairly large *U* value of 4.2 eV (Morgan and Watson, 2007) was required to empirically fit the calculated TiO_2_ bandgap to the experimental spectroscopic data.

In contrast, PBE0 severely overestimated the bandgaps of both the rutile and anatase phases, leading to underestimation of the dielectric constants. The self-consistent approach, which gave smaller α values than the value of 0.25 given by PBE0, resulted in smaller bandgaps and higher dielectric constants. In the case of the rutile phase, the scPBE0-determined bandgap of 3.25 eV is close to the electronic bandgap of 3.30 eV measured experimentally using photoelectron and inverse-photoelectron spectroscopies (PES/IPES) (Tezuka et al., 1994). This value further agrees with the G_0_W_0_ calculation results, with values ranging from 3.30 to 3.59 eV (Chiodo et al., 2010; Kang and Hybertsen, 2010; Landmann et al., 2012; Zhu and Gao, 2014; Sun et al., 2015). For the anatase phase, the scPBE0-determined bandgap of 3.75 eV also agrees with the G_0_W_0_ calculation results ranging from 3.56 to 3.86 eV (Chiodo et al., 2010; Kang and Hybertsen, 2010; Landmann et al., 2012; Patrick and Giustino, 2012; Zhu and Gao, 2014; Sun et al., 2015). Unfortunately, to the best of our knowledge, no experimental value for the electronic bandgap of anatase TiO_2_ is available in the literature, although the optical bandgap (which is known to be smaller than the electronic bandgap) was experimentally determined to be 3.47–3.53 eV (Reyes-Coronado et al., 2008; Baldini et al., 2017). It was thus concluded that scPBE0 most reliably predicts the structural and electronic properties of both TiO_2_ phases.

### Band Alignments of Rutile (110) and Anatase (101) Surfaces

**Figure 3:**
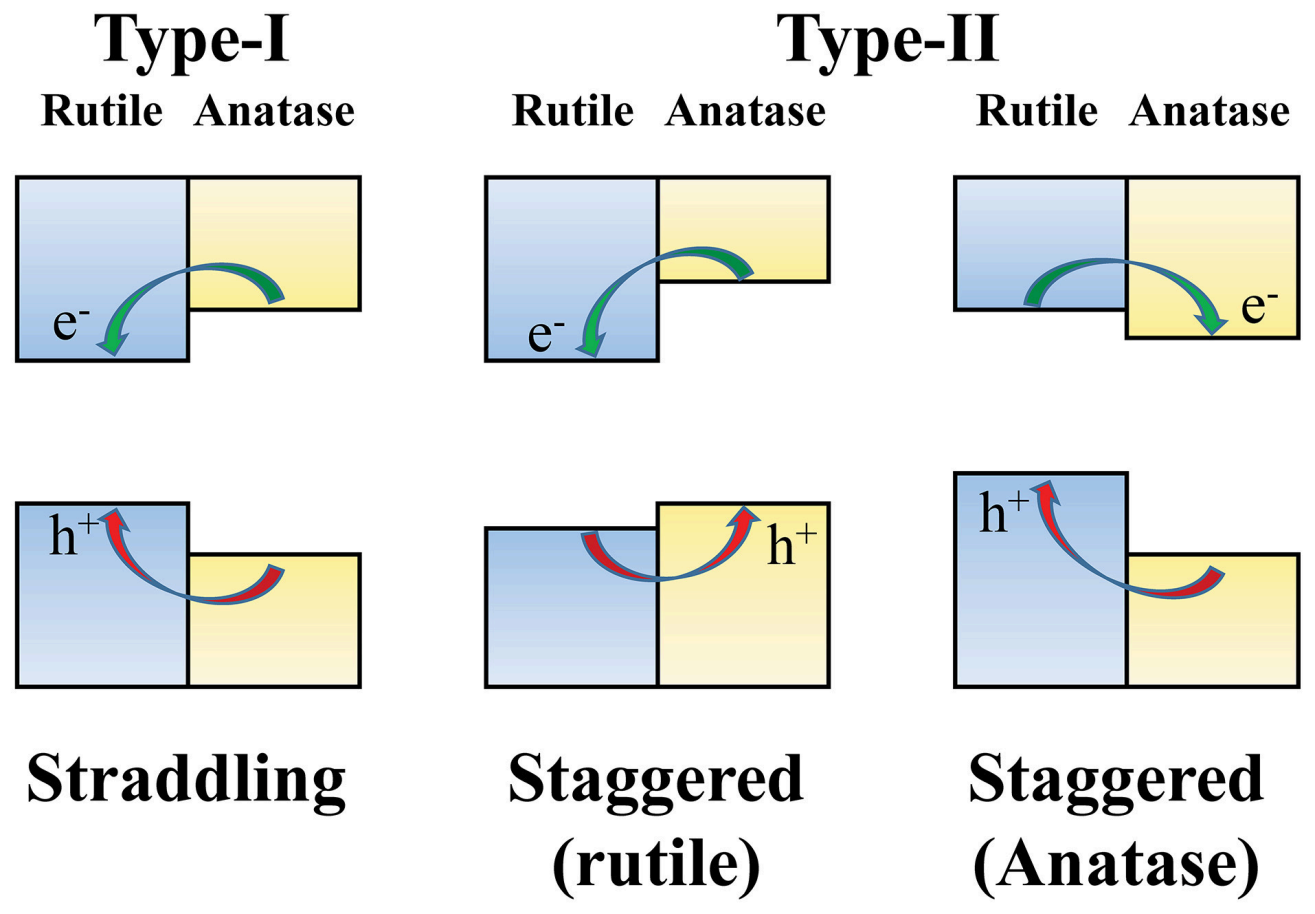
The proposed types of band alignment between rutile and anatase phases of TiO_2_. In type-I, also called “straddling type,” the excited electrons and holes are accumulated in the rutile phase. On the other hand, in type-II (“staggered type”), the two charge carriers are preferentially accumulated in different phases according to the sign of the relative band edge shifts.

For photocatalytic applications, an understanding of the surface band alignments in rutile and anatase TiO_2_ surfaces is important to elucidate the underlying physics of the charge carrier transfer between two phases. One open question in this regard relates to the relative band edge positions in rutile and anatase TiO_2_ surfaces (Scanlon et al., 2013). Two possibilities have been suggested, as illustrated in Figure 3: one is the straddling type (Kang et al., 2012), in which both the VBM and CBM of the rutile phase are located in the bandgap region of the anatase phase, and the second is the staggered type (Deák et al., 2011; Scanlon et al., 2013; Ju et al., 2014; Garcia et al., 2015), in which the VBM and CBM of the anatase phase are either up- or down-shifted from the VBM and CBM of the rutile phase.

**Figure 4:**
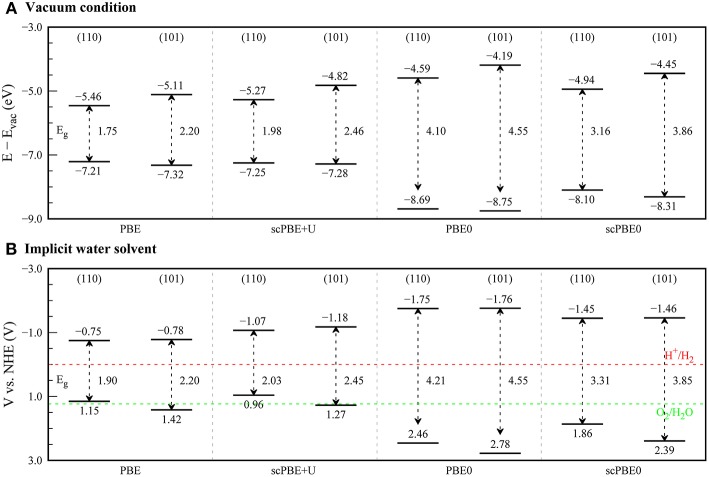
Band edge positions of rutile (110) and anatase (101) surfaces calculated using PBE, scPBE+*U*, PBE0, and scPBE0 methods. **(A)** Band edge positions in vacuum are aligned with respect to the vacuum potential, while **(B)** band edge positions in the aqueous electrolyte are aligned with respect to the normal hydrogen electrode (NHE) using the theoretical absolute electrode potential of 4.6 V. The reduction potential (H^+^/H_2_) and the oxidation potential (O_2_/H_2_O) for water splitting are presented as red and green dashed lines, respectively.

As such, we investigated the positions of the VBM and CBM of rutile and anatase TiO_2_ surfaces using various DFT methods. We investigated rutile (110) and anatase (101) surfaces, which are known to be the most stable surfaces for each phase. Before our main results, it is noteworthy to note about the effects of junction between the rutile and anatase TiO_2_. In its usual mixed-phase form of the TiO_2_ photocatalyst, the direct contact between two phases gives rise to the electron transfer from anatase to rutile phase, which affects to the band alignment and eventually to the photocatalytic activity (Kawahara et al., 2002). Not only the effects of the junction highly depend on the structure, especially at the interface, of the photocatalyst (Deák et al., 2011), but also, we need to model rutile-anatase interface to take account them. The latter, in terms of the computational cost, is a formidable task in our study with the hybrid functionals. As fundamental properties of their catalytic activities, we have focused on the band alignments between the rutile and anatase TiO_2_ without the junction effects by using the models of their surfaces separately. Figure 4A shows the surface band edge locations for each phase, aligned with respect to the vacuum level.

For the rutile (110) surface, PBE gave −7.21 and −5.46 eV for the VBM and CBM, respectively. When the *U* correction was included using scPBE+*U*, the VBM shifted downward slightly (−7.25 eV), whereas the CBM was up-shifted by a larger degree due to the increased band gap (−5.27 eV). The VBMs calculated using the hybrid functionals PBE0 (−8.69 eV) and scPBE0 (−8.10 eV) are much deeper than those obtained by PBE and scPBE+U, while the CBMs (−4.59 and −4.94 eV for PBE0 and scPBE0, respectively) are higher than those of the PBE and scPBE+U results.

Migani and co-workers calculated the band alignment of a rutile (110) surface using various approximated variants of quasiparticle GW methods (Migani et al., 2014). In their study, the positions of the VBMs were in the region between −7.3 and −8.8 eV, which is consistent with the experimentally derived VBM alignment. However, their band gaps were largely overestimated, resulting in a large up-shift of the CBMs in comparison to the experimentally derived energies. The position of the VBM from our scPBE0 method agrees reasonably well with the GW results of the same authors (Migani et al., 2014). Considering that the scPBE0-determined bandgap is also in good agreement with the experimentally determined electronic bandgap (as discussed above), it was concluded that the location of the CBM is also reliably predicted by the scPBE0 calculations.

Notably, the scPBE0 results support the straddling-type band edge alignment between the rutile (110) and anatase (101) surfaces. This is also consistent with the results of a previous DFT+*U* study by Zhang et al. (2015).

Motivated by the knowledge that most photocatalytic applications of TiO_2_ occur in aqueous medium (e.g., water splitting) (Ge et al., 2016), we further examined changes in the band edge positions in response to the effects of an aqueous electrolyte. We employed the Poisson-Boltzmann model to include the dielectric screening effect of the medium, as implemented in VASPsol code (Mathew et al., 2014; Mathew and Hennig, 2016). We considered an electrolyte consisting of an aqueous solution of monovalent anions and cations in 1M concentrations, by using a relative permittivity of 78.4 and a Debye length of 3 Å.

Figure 4B shows the band alignments of TiO_2_ aligned with respect to the theoretically determined absolute electrode potential of 4.6 V (Mathew and Hennig, 2016) instead of the vacuum level. We first compared the band edge positions with the reduction potential of H^+^/H_2_ (0.0 V vs. NHE) and the oxidation potential of O_2_/H_2_O (+1.23 V vs. NHE). From the PBE and scPBE+*U* results, the VBMs of the rutile (110) surface lie at a lower potential than the oxidation potential of O_2_/H_2_O, which is inconsistent with a previous experimental finding that TiO_2_ is one of the most-active water-splitting photocatalyst materials (Zhao and Liu, 2014; Ge et al., 2016; Gellé and Moores, 2017). In contrast, PBE0 and scPBE0 provided reasonable band edge positions that would energetically allow reduction and oxidation of the water molecule.

**Figure 5:**
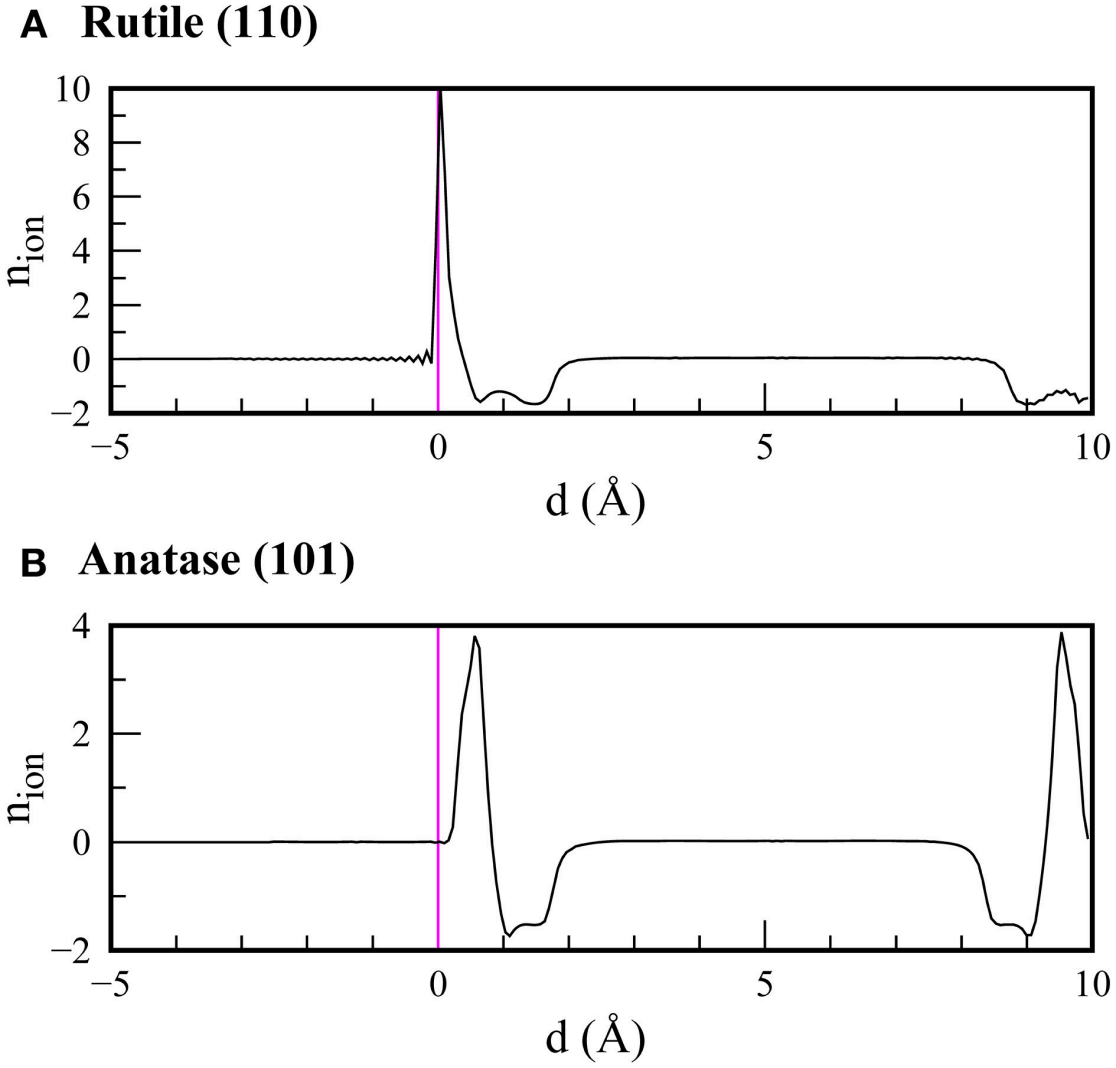
Bound charge distributions at **(A)** water-rutile (110) and **(B)** water-anatase (101) interfaces calculated using Poisson-Boltzmann theory coupled with scPBE0 calculations. The magenta lines depict the positions of the uppermost atoms in the surface slab of TiO_2_.

Interestingly, in comparison to the results in vacuum, we found that the CBM positions in the rutile and anatase surfaces became almost identical when the electrolyte solvation effect was taken into consideration. To elucidate the origin, we show the bound charge distributions at the water-rutile (110) and water-anatase (101) interfaces in Figure 5. We found that ions tended to accumulate at the water-TiO_2_ interface, forming a Stern-like layer at both interfaces. However, the water-rutile (110) interface showed a more profound tendency for Stern layer formation in comparison to the water-anatase (101) interface. Consequently, a stronger built-in potential imposed at the water-rutile (110) interface led to greater upward shifts of the band edge positions and CBM positions comparable between the rutile and anatase phases. This implies that the band alignments of TiO_2_ in an electrolyte can differ from those in vacuum, and that a transition from the straddling-type to the staggered-type band alignment may occur in an electrolyte medium, depending on the salt concentrations.

## Conclusion

In this study, we investigated the bulk properties of rutile and anatase phases of TiO_2_ as well as their surface electronic properties, using recently developed self-consistent variations of the hybrid functional and DFT+*U* methods, and with the conventional GGA(PBE) and PBE0 methods. The self-consistent hybrid functional method (i.e., scPBE0) demonstrated the best performance in predicting the bulk structural, elastic, and electronic properties of both phases of TiO_2_. In particular, the bandgaps of both the rutile and anatase phases of TiO_2_ were accurately described using the scPBE0 method, and the results were in good agreement with the experimental and/or the highly accurate GW results.

Based on the success of the scPBE0 method, we applied this method to calculations of surface slab models. We investigated the surface band alignment of rutile (110) and anatase (101) surfaces in a vacuum and in aqueous electrolyte, described using the Poisson-Boltzmann theory. Our scPBE0 method not only demonstrated the most reliable calculation of band edge positions, but also elucidated that the two surfaces form a straddling-type alignment with respect to each other in a vacuum. However, in the aqueous electrolyte, the solid-liquid interfacial field differently shifts the band edge locations, resulting in the CBMs of the rutile and anatase surfaces being located at nearly the same level. The difference was ascribed to the different tendencies for Stern-like layer formation at the water-rutile and water-anatase interfaces.

Our study suggests that the scPBE0 method, as an approximation of the GW method, can be a practical replacement for the computationally demanding GW calculations. It also suggests that the surface band alignment in an electrolyte can be different from that in a vacuum, and even implies that a transition from the straddling-type to the staggered-type band alignment might be possible by changing the salt concentration of the electrolyte. Our current work will not only provide a methodological suggestion for theoretical investigations on the electronic structures and band edge positions of large-scale systems, but will also provide useful insight into band alignment of two different TiO_2_ phases which can be helpful in the design of photoactive interfaces.

## Author Contributions

WK and HK conceptualized and designed the research. WK and MH performed calculations. WK, MH, and HK analyzed the results. WK wrote the original draft. WK, SL, EL, and HK reviewed and edited the manuscript. SL, EL, and HK supervised the research. WK and MH contributed equally to this work.

### Conflict of Interest Statement

The authors declare that the research was conducted in the absence of any commercial or financial relationships that could be construed as a potential conflict of interest.
